# Pancreatic Neuroendocrine Tumor (Pan-NET) Presented by Abdominal Pain: A Case Report and Literature Review

**DOI:** 10.3390/jcm12206617

**Published:** 2023-10-19

**Authors:** Matteo Regolo, Nicolas Cardaci, Clara Salmeri, Alfredo Laudani, Michele Colaci, Massimo Ippolito, Fabio Motta, Salvatore Magrì, Stefanie Parisi, Alfredo Gaetano Torcitto, Lorenzo Malatino

**Affiliations:** 1 Department of Clinical and Experimental Medicine, University of Catania, 95124 Catania, Italy; matteo.regolo.94@gmail.com (M.R.); alfred.laudani@gmail.com (A.L.); michele.colaci@unict.it (M.C.); 2 Academic Unit of Internal Medicine, Cannizzaro Hospital, 95126 Catania, Italy; 3 Nuclear Medicine Unit, Cannizzaro Hospital, 95126 Catania, Italy; ippolitomas@yahoo.it; 4 Pathological Anatomy Unit, Cannizzaro Hospital, 95126 Catania, Italy; fabio.motta@aoec.it; 5 Endoscopy Unit, Cannizzaro Hospital, 95126 Catania, Italy; salvo10ms@libero.it (S.M.); stefanie.parisi@aoec.it (S.P.); 6 Radiology Unit, Cannizzaro Hospital, 95126 Catania, Italy; alfre.84@katamail.com

**Keywords:** neuroendocrine tumors (NETs), pancreatic neuroendocrine tumors (Pan-NETs), endoscopic ultrasonography, EUS-FNA, EUS-FNB

## Abstract

A pancreatic neuroendocrine tumor (Pan-NET) is a rare neoplasm originating in the neuroendocrine system. Carcinoid syndrome occurs in approximately 19% of patients with functional Pan-NETs, typically when liver metastases occur. In this paper, we describe the case of a patient with a low-grade non-functional Pan-NET, but with a typical clinical presentation of carcinoid syndrome. An 81-year-old male was admitted to our Department of Internal Medicine at Cannizzaro Hospital (Catania, Italy) because of the onset of abdominal pain with nausea, loose stools, and episodic flushing. Firstly, an abdominal contrast-enhanced CT scan showed a small pancreatic hyper-vascular mass; then, a gallium-68 DOTATOC integrated PET/CT revealed an elevated expression of SSTR receptors. Serum chromogranin A and urinary 5-HIAA measurements were negative. We performed an endoscopic ultrasonography (EUS) by a fine-needle biopsy (EUS-FNB), allowing the immunostaining of a small mass (0.8 cm) and the diagnosis of a low-grade (G1) non-functional Pan-NET (NF-Pan-NET). Surgery was waived, while a follow-up strategy was chosen. The early recognition of Pan-NETs, although rare, is necessary to improve the patient’s survival. Although helpful to allow for immunostaining, EUS-FNB needs to be warranted in future studies comparing EUS-FNB to EUS-FNA (fine-needle aspiration), which is, to date, reported as the tool of choice to diagnose Pan-NETs.

## 1. Introduction

Neuroendocrine neoplasms (NENs) are enigmatic malignancies with an increasing incidence and prevalence [1,2,3,4]. Given their common morphological and immunophenotypical features, all these tumors arise from cells of the diffuse endocrine system.

NENs range from asymptomatic well-differentiated neuroendocrine tumors (NETs) to aggressive neuroendocrine carcinomas (NECs). In fact, nearly 80–90% of NENs are NETs, while the remaining 10–20% are carcinomas [5].

NETs can develop in any tissue of the body. The gastrointestinal tract and pancreas are the most common sites of origin, accounting for approximately 60% of the primary sites [6], followed by the lungs and other sites.

About 40% of NETs can release hormones responsible for symptoms, depending on the secreted hormone. Carcinoid syndrome is characterized by episodic flushing and diarrhea, due to various vasoactive substances (serotonin, histamine, and other amines) released into the systemic circulation [7].

Non-functional NETs may often present with subtle and sporadic symptoms, sometimes with gastrointestinal bleeding, abdominal pain, bowel obstruction, or unexplained weight loss [8].

Treatment and prognosis depend on the grade and stage of the tumor. NETs diagnosis is frequently late, along with symptoms related to hormone hypersecretion, often after metastases occurs in the liver, where bioactive substances fail to be inactivated. An early diagnosis and recognition are necessary to improve the patient’s survival, which has not significantly changed over the last 30 years [9].

In this paper, we present a case of a pauci-symptomatic pancreatic neuroendocrine tumor in a patient with an unspecific clinical presentation (abdominal pain) and mild additional symptoms (nausea and loose stools). This was the occasion for a narrative review of the literature on the diagnosis and management of pancreatic neuroendocrine tumors (Pan-NETs).

## 2. Case-Report

In May 2023, an 81-year-old man was admitted to the Department of Internal Medicine at Cannizzaro Hospital (Catania, Italy) because of the onset of abdominal pain, especially in the lower abdominal quadrants, with nausea and loose stools (<3 times/day).

The patient’s past medical history included arterial hypertension, type-2 diabetes mellitus, peripheral artery disease (PAD), obesity, hypothyroidism, and depressive syndrome. In the past six months, he complained of abdominal distension and changes in bowel habits (loose stools). There was no relevant family history. He was taking levothyroxine, insulin according to HGT, lansoprazole, acarbose, ezetimibe/simvastatin, and furosemide. He denied the anamnestic consumption of uncooked meat, fish, or unpasteurized dairy products.

On admission, he had no fever, arterial hypertension (177/76 mmHg), had a normal heart rate (86 bpm), glycemia of 102 mg/dL, and normal SaO_2_ in room air (98%); he presented no sensorium alterations. A physical examination revealed abdominal distension, with colic pain on deep palpation and hypoactive abdomen sounds. Mucous membranes were normally hydrated. The bedside FAST (Focused Assessment with Sonography in Trauma) scan did not detect peritoneal fluid. The digital rectal examination showed blood traces.

Laboratory tests were performed, showing an increase in serum CRP (17.9 mg/dL), moderate leukocytosis, moderate renal dysfunction (serum Cr: 1.33 mg/dL, eGFR: 54 mL/min/1.73 m^2^), normal serum potassium (3.6 mEq/L), sodium (139 mEq/L) and chloride (100 mEq/L), mild metabolic acidosis (pH: 7.33, HCO_3_: 21 mmol/L, pCO_2_: 42 mmHg), and serum procalcitonin < 0.2 ng/mL. Infectious causes of diarrhea were excluded by microbiological and chemical fecal examinations. An abdomen X-ray excluded bowel obstruction or perforation. Moderate intravenous fluid repletion was administered.

A few hours after admission, the patient experienced transient states of agitation, with uncontrolled crying spells and temper tantrums. Due to his past medical history of untreated depression, anxiolytic and antipsychotic therapies were prescribed, followed by poor efficacy. During this altered emotional status, a flushing episode was observed in the face and neck.

**Figure 1:**
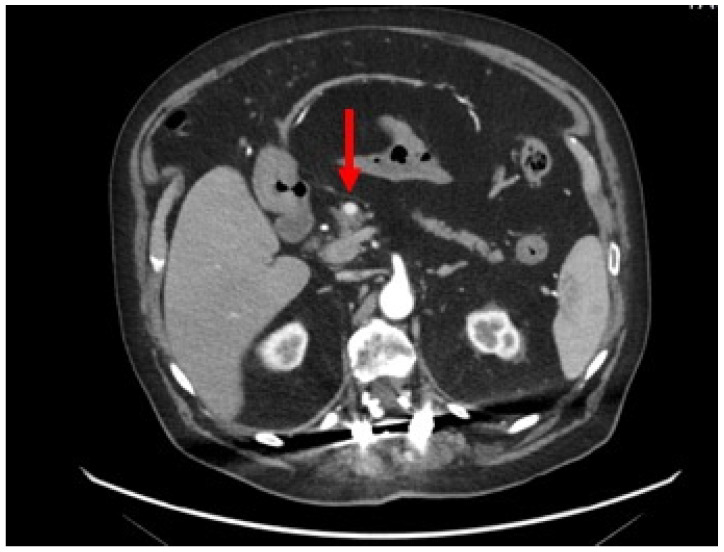
Contrast-enhanced abdominal CT scan: axial section showing a homogeneous and hypervascular mass of 8 mm (red arrow) on the arterial phase.

A contrast-enhanced abdominal CT scan revealed a pancreatic hypervascular small mass (8 mm) (Figure 1).

**Figure 2:**
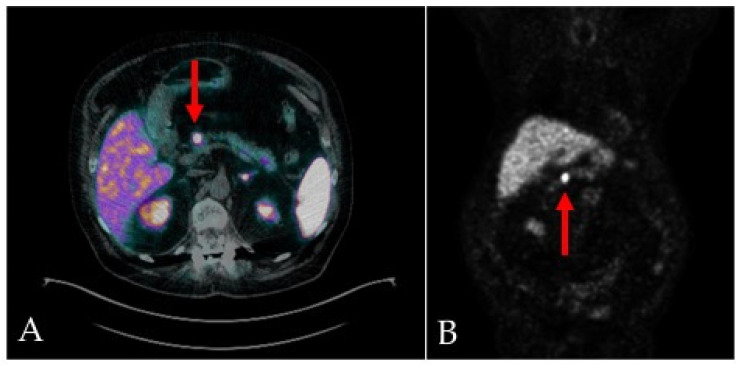
^68^Ga-DOTA-TOC integrated PET/CT scans, transaxial (**A**) and MIP (**B**), show focal and intense uptake in the primary pancreatic lesion (red arrows), with an elevated expression of SSTR2/5 somatostatin receptors.

On the fifth day of admission, given the suspicion of a pancreatic neuroendocrine tumor (Pan-NET), a gallium-68 DOTATOC integrated PET/CT was performed (Figure 2), confirming a small mass between the head and body of the pancreas, with an elevated expression of SSTR2/5 somatostatin receptors. No other sites of disease were detected.

The serum chromogranin A (CgA) measurement was within the normal range (98.0 ng/mL, normal values < 101.9 ng/mL); we also performed a urine 5-HIAA test (urinary 5-HIAA: 1.6 mg/24 h; normal values: 1.0–8.2 mg/24 h).

A progressive recovery was observed, with no further abdominal pain. In accordance with the remission of symptoms and the normal laboratory values, the patient was discharged with the prescription to undergo an endoscopic ultrasonography with a fine-needle biopsy (EUS-FNB) for targeted diagnostic and therapeutic management.

**Figure 3:**
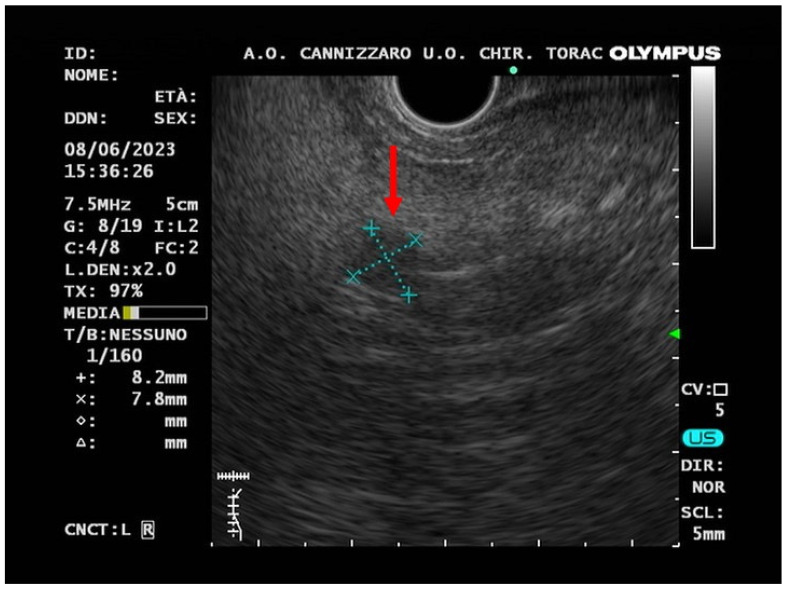
Endoscopic ultrasound (EUS) image (red arrow) of a small hypo-echogenic lesion with a regular margin and a major axis of 8.9 mm.

In June 2023, an EUS-FNB, performed with a 22-gauge Acquire needle (Boston Scientific, Marlborough, MA, USA) using a slow-pull technique, visualized the presence of an oval hypo-echogenic mass, with a major axis of 8.9 mm (Figure 3), which was sampled for the cyto-histological examination.

**Figure 4:**
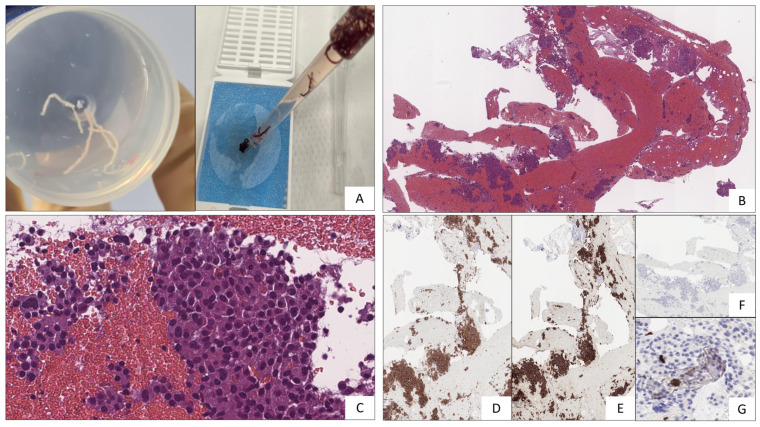
(**A**) Microcores of sample tissue. (**B**) Abundant blood and entrapped epithelial elements of pancreatic tissue stained with Hematoxylin–Eosin. (**C**) Epithelial cells, with a granular cytoplasm and nuclei with thickened chromatin (Hematoxylin–Eosin staining). (**D**) Chromogranin A (5H7 clone, immunohistochemical staining). (**E**) Synaptophysin (27G12 clone, immunohistochemical staining). (**F**) Serotonin (YC5/45 clone, immunohistochemical staining). (**G**) Ki67 (MM1 clone, immunohistochemical staining).

Histological and immunohistochemical examinations confirmed the suspicion of Pan-NET (stage WHO G1, well-differentiated, synaptophysin positive, CgA positive, Ki67 1%) (Figure 4). The fine-needle biopsy allowed us to obtain microcores of the sample tissue (Figure 4A). Then, using a pipette, the microcores were picked up to be treated as a traditional biopsy. The microcores were composed of abundant blood and entrapped epithelial elements of pancreatic tissue (Figure 4B). A monomorphic population of epithelial cells, in solid sheets or small nodules, with a granular cytoplasm and nuclei with thickened chromatin was also observed (Figure 4C). Immunochemistry, performed with a Bond-Leica immunostainer, revealed positivity for neuroendocrine markers, such as chromogranin A (Figure 4D) and synaptophysin (Figure 4E), while that of serotonin was negative (Figure 4F). The absence of mitosis and necrosis, together with a low Ki-67 index (Figure 4G), allowed us to determine a low-grade neuroendocrine neoplasm.

In keeping with the current guidelines, these findings suggest the diagnosis of a low-grade (G1) non-functional pancreatic neuroendocrine tumor (NF-Pan-NET) (well-differentiated neoplasm, absence of mythosis, Ki67 ≤ 2%) [10]. This definition of “non-functional”, based only on negative hormone tests, was finalized to a categorical distinction between “secreting” and “non-secreting” tumors, although it underestimated the importance of the clinical presentation.

After the evaluations of the stage, grading, symptoms, and comorbidities, a conservative approach of watchful waiting was chosen by the surgeon, with a radiological follow-up session after one year. We scheduled a clinical follow-up session in order to keep the symptoms under observation.

## 3. Review of the Literature

Neuroendocrine neoplasms (NENs) are heterogenous neoplasms arising in the secretory cells of the diffuse neuroendocrine system, the so-called APUD (Amine Precursor Uptake and Decarboxylation) System [4]. Characterized by amine and neuropeptide hormone production with dense vesicles, these neuroendocrine cells are specialized to receive neuronal inputs and consequentially release message peptides into circulation for the regulation and modulation of cell proliferation, growth, and development. NENs are distinguished from pheochromocytomas and paragangliomas (neuroendocrine non-epithelial neoplasms) by the expression of keratin in the former ones, given their epithelial origin [11].

Neuroendocrine tumors (NETs) represent only 0.5% of all malignant conditions and 2% of all malignant tumors in the gastrointestinal tract [12]. Given the continued update in the classification of NENs, these epidemiological data are continuously evolving. The prevalence of NETs ranges between 2.5 and 8.35 cases per 10,000, with a recent increase in their incidence rates [1,2,3,4,13,14,15,16], probably due to imaging improvement, leading to an earlier and more frequent diagnosis of the disease [6].

In the 2019 WHO classification of tumors of the digestive system [17], NENs are divided into well-differentiated neuroendocrine tumors (NETs) and poorly differentiated neuroendocrine carcinomas (NECs), based on their molecular differences. In addition, “mixed neuroendocrine–non-neuroendocrine neoplasms” (MiNENs) are better characterized, according to the simultaneous presence of both neuroendocrine and non-neuroendocrine components, typically poorly differentiated (Table 1).

The most frequent primary sites are the gastrointestinal tract (61%), lung (25%), and about 14% remains of an unknown origin [18]. A total of 12 to 22% of patients are metastatic at presentation [6].

Recently, abdominal pain was reported as an unspecific symptom of a small bowel NET [19]. Our case report resembled that very recently described by Daraghmeh et al. [19]; although, in our patient, we found a Pan-NET.

The 2019 WHO classification [17] provided an improved system for determining prognoses and treatments, appliable to all NENs, replacing the previous classification based on cell embryologic origin (foregut, midgut, and hindgut) [20]. In contrast to the 2017 WHO classification of tumors of endocrine organs [21], the last classification included pancreatic tumors in gastroenteropancreatic NENs (GEP-NENs) [17].

Gastroenteropancreatic tumors (GEP-NETs) are most commonly located in the gastric mucosa, the small intestine, the rectum, and the pancreas [4,22]. While a subset of NENs is functional (40%), presenting with characteristic endocrine-related symptoms, most of them are non-functional and do not present with symptoms until later stages.

The distant metastases of NF-PNETs are often found at the time of diagnosis, because symptoms of NF-PNETs develop in an advanced stage. Due to these characteristics, NF-PNETs are usually incidentally diagnosed, like GEP-NETS, thanks to the development of imaging techniques, able to also identify very small lesions. In our patient, the presence of flushing, diarrhea, and neuropsychiatric symptoms, suggesting carcinoid syndrome, was unrelated to a biochemical elevation of hormonal levels. As a matter of fact, small PNETs without metastases can often remain asymptomatic until they reach a significant dimension, or can present with unspecific symptoms, such as abdominal pain, weight loss, anorexia, and nausea.

Up to 90% of Pan-NETs are hormonally silent, a behavior affecting the prognosis as compared to functioning neoplasms, probably because of a late diagnosis [23].

Pan-NETs may produce a large variety of hormones, such as insulin, gastrin, glucagon, vasoactive intestinal peptide (VIP), serotonin, somatostatin, and others [24]. By contrast, non-functional Pan-NETs, without hormone overproduction, may present with unspecific symptoms, such as abdominal pain, weight loss, diarrhea, and gastrointestinal bleeding [8,25]. Most Pan-NENs are sporadic, whereas a minority are inherited, associated with type-1 multiple endocrine neoplasia (MEN-1), von Hippel–Lindau syndrome (VHL), tuberous sclerosis, or neurofibromatosis.

Functional pancreatic neuroendocrine tumors, associated with a variety of clinical syndromes, include [26] insulinomas, the most common functional Pan-NETs; gastrinomas, or Zollinger–Ellison syndrome; pancreatic polypeptide-secreting tumors; VIPomas, or Verner–Morrison syndrome; glucagonomas, exclusively localized in pancreas; and somatostatinomas, the least common NETs.

Carcinoid syndrome is a paraneoplastic syndrome that occurs because of the release of bio-active substances, predominantly serotonin (5-HT), but also histamine, bradykinin, prostaglandins E and F, and tachykinins [27]. The typical symptoms are flushing and diarrhea. Wheezing, palpitations, breathlessness, abdominal pain, telangiectasias, and neuropsychiatric symptoms can also be associated with carcinoid syndrome [27,28]. Recently, Halperin et al. [29] demonstrated in a population-based analysis conducted on the American “Surveillance, Epidemiology, and End Results Medicare” database that 19% of patients with NETs had carcinoid syndrome. In patients harboring Pan-NETs, carcinoid syndrome is even more rare, accounting for approximately 1% [13].

The diagnosis of GEP-NENs is performed on the basis of a tissue histological examination [30]. Radiological and functional imaging is used to evaluate disease extension (staging) and assess the response to therapy, as well as to localize the primary site. Laboratory tests play a diagnostic role only in carcinoid syndrome and hormone-specific syndromes (gastrinomas, insulinomas, and glucagonomas), although the assay of either circulating or urinary hormones fail to be highly sensitive and specific, sometimes because blood sampling and urine collection are not performed closely to the occurrence of typical symptoms.

The current WHO classification emphasizes the role of histological examinations in surgically removed neoplasms, in order to establish the morphological characteristics and grading [17]. Three grades (G1, G2, and G3) are described for GEP-NETs, based on the proliferation activity assessed by the mitotic rate and Ki67 proliferation index [31,32]. For a more specific diagnosis, together with the morphology and grading, the immunohistochemical staining of chromogranin A (CgA) and synaptophysin should be assessed as biomarkers of neuroendocrine tumors.

Although the WHO histological classifications are specifically intended for surgically removed NENs [10,17], recent studies have investigated the role of endoscopic ultrasound-guided fine-needle aspiration (EUS-FNA) and fine-needle biopsy (EUS-FNB) for the pre-operative evaluation and management of pancreatic NETs (Pan-NETs) [33,34,35,36,37,38,39,40,41,42]. Despite the data for the grading agreement between EUS-FNA and surgical specimens highlighting the significant rate of under- or over-grading [41,42,43,44,45,46,47], the recent introduction of needles for EUS-guided fine-needle biopsies (EUS-FNBs), as for our patient, changed the scenario [37,38]. An EUS-FNB, in fact, allows us to obtain tissue samples on which immunohistochemical examinations can be easily performed, to evaluate the Ki67 proliferation index [39,40,41,42,43,44,45]. As a matter of fact, in patients harboring Pan-NETs smaller than 2 cm, the management remains still controversial, especially for asymptomatic and non-functional Pan-NETs [46,47,48,49]. Endoscopy with a biopsy is already the gold standard for diagnosing NENs of the stomach, duodenum, and colorectum [50,51]. In the diagnosis of pancreatic NENs, EUS is particularly useful in detecting the nature of small lesions. The introduction of EUS-FNB can then overcome the interpretative limits of EUS-FNA, therefore allowing the early characterization of tumors where surgery would destroy healthy tissue [39,40]. However, further prospective, randomized studies are needed to validate these approaches in the specific setting of Pan-NETs [30,52].

The surgical treatment of patients with small low-grade non-functional pancreatic neuroendocrine tumors (<20 mm) is still under debate, according to the ENETS guidelines. In this respect, Sugawara et al. [53], in a recent metanalysis, demonstrated that a surgical resection was recommended in patients with nonmetastatic NF-PNETs measuring between 1.1 and 2.0 cm; alternatively, those patients with a smaller lesion (<1 cm) showed greater prognostic benefits with a conservative approach. JNETS [54] suggests a follow-up strategy, with imaging every 6–12 months of asymptomatic tumors <1 cm without metastases. Moreover, Sadot et al. [55] further reported that, among 104 patients with small, asymptomatic Pan-NETs undergoing non-operative management, no patient developed evidence of metastases or died because of the tumor after a median follow up of 44 months. Several studies suggested that a surgical intervention may not be warranted for very small Pan-NETs, especially in elderly individuals [56,57,58]. It is noteworthy however that all these data were obtained from a population much younger (median age: 60–65 years) than our patient (81 years old).

Paik et al. [59] suggested that patients with Pan-NETs smaller than 1 cm could be managed by observation alone, while Pan-NETs > 1 cm should undergo EUS-FNBs to obtain grading and Ki67 immunostaining, to characterize the tumor according to the WHO classification.

To investigate Pan-NETs, several imaging techniques can be performed, including computed tomography (CT), magnetic resonance (MRI), ultrasonography, and functional imaging with scintigraphy and positron emission tomography (PET). The optimal choice of imaging modality depends on the location of primary and metastatic lesions [60].

Endoscopic ultrasonography (EUS) has become the gold standard technique to evaluate pancreatic neuroendocrine lesions [4,30,61,62]. On an EUS, Pan-NETs typically appear as well-defined, round, hypoechoic, homogenous vascular lesions [63]. As in our case report, the EUS allowed the accurate localization of Pan-NETs, which was crucial for surgical interventions. As mentioned before, the EUS allows the cyto-histological confirmation of neuroendocrine tumors through guided tissue acquisition for histological procedures [33,34,35,36,37,38,39,40,41,42].

The functional imaging of GEP-NENs is based on the typical expression of somatostatin receptors (SSTRs) by neuroendocrine cells [64]. In the past, functional studies were performed with ^111^indium pentetreotide scintigraphy (Octreoscan^®^); in recent years, PET/CT with somatostatin analogs tracked with gallium-68 (^68^Ga-SSA PET/CT) has become the modality of choice for SSTR imaging [10,65,66]. Functional imaging is indicated for staging, the localization of the unknown primary tumor in patients with established neuroendocrine metastases, the in vivo demonstration of SSTR expression in neuroendocrine cells (for therapeutic planning), as well as the extent of disease after treatment. The most common somatostatin analogs used in clinical practice are ^68^Ga-DOTA-Tyr3-octreotide (^68^Ga-DOTA-TOC), ^68^Ga-DOTA-Tyr3-octreotate (^68^Ga-DOTA-TATE), and ^68^Ga-DOTA-Nal3-octreotide (^68^Ga-DOTA-NOC). The mean sensitivity of ^68^Ga-DOTA-SSA PET/CT for the diagnosis of Pan-NETs was 92%, while the specificity was 83% [67,68]. In advanced, fast-growing G2 and G3 NENs, especially if receptor negativity was evident at ^68^Ga-SSA PET/CT, ^18^FDG-PET/CT could be considered in the diagnostic approach [69,70]. The detection of Pan-NETs with functional imaging can be affected by the physiological uptake, especially in the uncinate process, therefore suggesting morphological imaging together with histological confirmation as a specific diagnostic process [71]. However, it still remains under debate whether the combined use of ^18^FDG-PET/CT and the ^68^Ga-DOTA-TOC peptide can improve the diagnostic performance of NENs [70]. Of note, a recent retrospective study [72] confirmed the suggestion of the combined use of 68Ga-DOTA peptides and 18F-FDG as radiotracers for a dual-tracer PET/CT to better evaluate tumor aggressiveness before surgery, especially for small masses of doubtful interpretation, when a metabolic confirmation of biopsy grading is needed [73].

At present, the biochemical diagnosis of NENs has been downsized due to the high proportion of non-functioning NENs. Considering the high rates of false-positive and heterogeneous serum determinations, chromogranin A (CgA) should be used in patients with an already documented diagnosis of NEN, in order to establish the treatment response or during the follow up [74,75]; although, the results are less sensitive for the primary diagnosis. On the other hand, neuron-specific enolase (NSE) is considered an unreliable diagnostic biomarker for NETs, due to its low sensitivity and specificity, while no evidence is available regarding its role in the follow up [76].

Laboratory tests for specific biomarkers (gastrin, insulin, glucagon, VIP, and 5-HIAA) are still important tools for certain clinical syndromes. 5-hydroxyindoleacetic acid (5-HIAA), detected in a 24 h urine collection using optimal conditions for the assay, is the specific tumor marker of carcinoid syndrome. 5-HIAA demonstrated a diagnostic sensitivity of 70%, with a specificity of 90% [77]. It is not recommended to use 5-HIAA as a screening test in the presence of diarrhea. Instead, it should be used in patients diagnosed with NENs to confirm carcinoid syndrome and assess its response to therapy [10,77].

Circulating tumor cells, circulating tumor DNA, circulating micro-RNAs, and NETest (the simultaneous measurement of 51 neuroendocrine-specific marker genes in the peripheral blood) are novel biomarkers under validation for NENs. However, this test is not widely available and needs further validation [78].

The treatment of Pan-NENs depends on the functionality, localization, dimension, and disease progression of the tumor. In most cases, surgical resection is the appropriate curative treatment in functioning pancreatic NET syndromes without metastases [49,54]. As for NF-Pan-NETs, surgical treatment, when feasible, is the gold standard [46,79,80], even if, as previously mentioned, a surgical intervention may not be warranted for very small Pan-NETs (<1.0 cm), especially in elderly individuals [53,54,55,56,57,58]. Surgical options include simple enucleation, central pancreatectomy, distal pancreatectomy with or without a splenectomy, and pancreatoduodenectomy (Whipple’s operation), depending on the tumor’s location. Moreover, radiofrequency ablation and trans-arterial chemoembolization are used for liver metastases.

When a macroscopic curative resection is unfeasible, medical treatment is indicated to control hormonal symptoms in F-Pan-NETs and to reduce the tumor’s growth. Since the majority of GEP-NETs express somatostatin receptors (SSTRs), somatostatin analogs are used in F-Pan-NETs, together with adequate treatments for specific clinical syndromes (for example, PPi in ZES) [81]. For tumor growth control, somatostatin analogs, molecular-targeted drugs, and cytotoxic anticancer agents are indicated, regardless of functionality [82]. SSAs are the first choice when a positive expression of SSRT is confirmed. The use of lanreotide and octreotide long-acting release (LAR) were already proven to be effective in reducing a tumor’s progression [81,83,84]. Recently, Wolin et al. [85] reported that the use of pasireotide, a novel SSA, despite a more extensive antiproliferation effect, was associated with more frequent adverse events. Targeted therapy, with everolimus and sunitinib, chemotherapy, and peptide-receptor radionuclide therapy (PRRT) should generally be reserved for SSA-refractory cases [49].

## 4. Discussion

Our case report described an old patient with an extremely rare pancreatic neuroendocrine tumor (Pan-NET), diagnosed in the presence of unspecific gastrointestinal symptoms and skin flushing. This observation is even rarer in old people. Despite the symptoms suggesting carcinoid syndrome, the tumor was well-differentiated and localized in the pancreas without liver metastases. This presentation is extremely rare, with few cases reported in the literature [86,87,88]. Biochemical testing for serum CgA and urinary 5-HIAA resulted negative. As previously emphasized, laboratory biomarkers were recently downsized due to the high rates of false positivity and their pharmacological interference, leading to low sensitivity and specificity [74,75,76,77,78,89].

We confirmed the Pan-NETs diagnosis through a contrast-enhanced CT, followed by functional imaging with a gallium-68 DOTATOC integrated PET/CT. We decided to perform an EUS-FNB to test immunostaining for the main markers of Pan-NETs and obtain grading. EUS-FNB confirmed the diagnosis of well-differentiated, low-grade (G1) Pan-NET (CgA+, Synaptophysin+, Ki67 1%).

The association of NETs and carcinoid syndrome occurs in approximately 19% of patients [27]. Except for patients with primary ovarian or bronchial neuroendocrine tumors, the evidence of carcinoid syndrome develops when metastases have occurred [26,28]. As a matter of fact, serotonin-producing Pan-NETs account for 0.58–1.4% of all Pan-NETs [90,91]. Only a few cases have been previously reported for Pan-NETs without liver metastases presenting with carcinoid syndrome [87,92]. Some patients with neuroendocrine tumors showed symptoms of flushing with low or normal levels of 5-HIAA [93,94]. Negativity for the immunostaining of serotonin found in our tumor biopsy, while in keeping with the normal values of 5HIAA, may further support the notion that levels of circulating hormones can increase only in the presence of liver metastases [29]. Our patient experienced carcinoid symptoms (diarrhea, flushing, and unresponsive depression) in the absence of documented liver metastases and with negative serum CgA and normal urinary 5-HIAA levels. The guidelines clearly show that negative hormone measurements define NETs as “nonfunctional”, even if presenting with suggestive symptoms or positive hormonal expressions in NET cells on immunohistochemical staining [49]. This may not always true, as can be observed in our case-report, as well as in few other reports [87,92].

It remains unclear why symptoms resembling carcinoid syndrome developed in our patient, with no evidence of any increase in hormone levels. It may well be that a possible, sudden, and transient hormone increase in the circulation failed to be detected. Otherwise, some to date unknown mechanisms might have been responsible for the abdominal pain, diarrhea, and flushing, all together causing us to consider alternative diagnoses regarding bowel diseases, which were excluded by the contrast-enhanced CT scan in our patient. In the presence of this discrepancy between the presence of symptoms and hormone negativity however, our case report emphasized that the clinical presentation should not be disregarded as a presentation of carcinoid-like syndrome, therefore leading to a complete diagnostic work-up for NETs.

Therefore, despite this, the Pan-NET of our patient should be defined as non-functional according to the guidelines [49], because the hormone values were within the normal range; our case report demonstrated that the imaging and histological examinations were useful in the diagnostic work-up of a Pan-NET associated with symptoms of carcinoid syndrome. As we reported, performing an EUS-FNB and assessing the cyto-histological features can help characterize the Pan-NET. Of note, we again underscore the concept that Pan-NET occurrence without metastases in old patients is very rare.

A Pan-NET < 1.0 cm can occur in very old people, without metastases, as in our case report; although, the median age was between 61 and 65 years in a recent metanalysis [50]. A surgical resection in these cases is not warranted. On the contrary, for Pan-NETs between 1.0 and 2.0 cm, surgical resections provided a better survival outcome, but in patients younger than 65 years old, without comorbidities.

The novelties of our case report can be highlighted as follows: (1) the symptoms of carcinoid syndrome can be shown in a Pan-NET < 1.0 cm occurring in very old people, without metastases, and with no evidence of an increase in circulating hormones, in agreement with the negativity of immunostaining for serotonin shown in tumor tissue. To date, the median age range was much lower [52]. (2) The categorical distinction of “functional” and “nonfunctional” NETs suggested by the guidelines [49] on the basis of hormone positivity and clinical presentation can help present Pan-NETs with no evidence of hormone release, as in our case report, thus underscoring the concept that the physician should take into account the possibility that an atypical pattern of apparently “non-functional” Pan-NETs may occur, although rarely. (3) In our patient, the EUS-FNB offered the opportunity of obtaining additional data regarding the immunostaining of the small Pan-NET; although, to date, an EUS-FNA is recognized as the method of choice in the multidisciplinary diagnostic approach of occult primary NETs, as recently reported by Rossi et al. [62]. Further evidence is needed to understand whether an EUS-FNB, as reported by a recent multicenter study [53], can provide physicians with additional details of the diagnostic workup of Pan-NETs.

In conclusion, this case report contributes to the understanding of the clinical spectrum of Pan-NETs, particularly in elderly patients, and highlights the potential challenges in decision making when treating patients with indolent neoplasms, as well as well-differentiated Pan-NETs surgically or even medicinally. It also highlights the role of advanced diagnostic techniques, such as EUS-FNB, in characterizing P-NETs.

## Figures and Tables

**Table 1:** WHO classification (2019) and grading criteria for gastroenteropancreatic neuroendocrine neoplasms (GEP-NENs) [17].

	Differentiation	Grade	Mitotic Rate (Mitoses/2 mm^2^)	Ki-67 Index
NET, G1	Well differentiated	Low	<2	<3%
NET, G2		Intermediate	2–20	3–20%
NET, G3		High	>20	>20%
NEC, small-cell type	Poorly differentiated	High	>20	>20%
NEC, large-cell type			>20	>20%
MiNEN	Well or poorly differentiated	Variable	Variable	Variable

## Data Availability

The data presented in this study are available on request from the corresponding author.

## Abbreviations

APUD	Amine precursor uptake and decarboxylation
CgA	Chromogranin A
CHD	Carcinoid heart disease
Cr	Creatinine
CT	Computed tomography
CRP	C-reactive protein
eGFR	Estimated glomerular filtration rate
ENETS	European neuroendocrine tumor society
EUS-FNA	Endoscopic ultrasound with fine-needle aspiration
EUS-FNB	Endoscopic ultrasound with fine-needle biopsy
FAST	Focused assessment with sonography in trauma
^68^Ga-DOTA-NOC	Gallium-68-DOTA-Nal3-octreotide
^68^Ga-DOTA-TATE	Gallium-68-DOTA-Tyr3-octreotate
^68^Ga-DOTA-TOC	Gallium-68-DOTA-Tyr3-octreotide
GEP-NEN	Gastroenteropancreatic neuroendocrine neoplasm
GEP-NET	Gastroenteropancreatic neuroendocrine tumor
HGT	Hemo-glucose test
5-HIAA	5-hydroxyindoleacetic acid
5-HT	Serotonin
INSM1	Insulinoma-associated protein 1
JNETS	Japanese neuroendocrine tumor society
MEN-1	Multiple-endocrine neoplasia 1
MiNEN	Mixed neuroendocrine–non-neuroendocrine neoplasm
MRI	Magnetic-resonance imaging
NF-Pan-NET	Non-functional pancreatic neuroendocrine tumor
NEC	Neuroendocrine carcinoma
NEN	Neuroendocrine neoplasm
NET	Neuroendocrine tumor
NSE	Neuron-specific enolase
Pan-NET	Pancreatic neuroendocrine tumor
PAD	Peripheral artery disease
PET	Positive-emission tomography
PPi	Proton pomp inhibitor
PPRT	Peptide receptor radionuclide therapy
SaO2	Oxygen saturation
SSA	Somatostatin analog
SSTR	Somatostatin receptor
VHL	von Hippel–Lindau syndrome
VIP	Vaso-active intestinal peptide
WHO	World Health Organization
